# Do We, in Our Practice, Do the Best in Our Power for Our Patients?

**Published:** 1890-12

**Authors:** H. C. Raymond

**Affiliations:** Detroit


### Do We, in Our Practice, do the Best in Our Power for Our Patients?

BY H. C. RAYMOND, D.D.S., DETROIT.

Read before the Detroit Dental Society, Oct. 13th, 1890.

*Mr. President and Gentlemen:*—I have no case in practice to present to you which would be of any special interest, nor have I any new theories or ideas to expound. The ground on which I am going to venture a few remarks may, I suppose, in a sense be termed ethical. I think I hear some of you say that we have enough of ethics at our State meetings. Well, perhaps we have and for that reason I won't mention ethics, though my remarks may sound rather strongly of that much vexed subject.

Being a young practitioner I trust I may not be considered presumptuous for venturing on ground on which it would appear that you, who have grown gray in the profession, are much more at home than the beginner, and are rather able to teach him how to tread it and conduct himself while on it. For that reason I am inclined to wish that more young men were present, for what I have to say would appear to apply to the young members in our profession, still, as some of the evils to which I shall refer exist in practices of some of the older men, I do not deem them entirely out of place, and my remarks may cause discussion and bring out suggestions and ideas that may be beneficial to all.

From observation in practice the question has often suggested itself to me, do we, in our practice, do the best in our power for our patients? That question I will endeavor to answer in my remarks, as well as asking other questions.

During my short period of practice in this city I have had many points brought to my notice both from remarks of my patients and the conditions of their mouths, which has caused me like the parrot to do a heap of thinking and to reflect as to whether we are all conducting our practices for the individual interest of our patients as well as our brother dentists.

When a patient presents herself for our service, not knowing what is required but relying on our judgement and ability to do what is necessary and best for her, I think we are not faithful to our calling if we do not put our skill to the best possible advantage and give the patient the full benefit of our knowledge. For instance a patient comes in to have a tooth attended to and does not know that any other teeth need attention, is it not our duty to make an examination of the mouth, find out its condition and inform the patient of the necessity of having other work done? for in nearly every case it will be found that there is much more work to be done than the patient has any idea of. By doing this, many more teeth might be saved, and better operations be done than if the disease was allowed to make its progress. And if it is our duty to do this when we are not requested, how much more is it our duty to make a thorough examination when asked to do so by the patient. I think it nearly criminal to carelessly look over the mouth and allow a patient to leave the office satisfied that her molars are all right and need no further attention when wholesale destruction is often taking place. Yet from my observation cases are not rare in which such carelessness is exhibited. Whether it is really due to carelessness or because the operator has more work than he can properly attend to I can not say, there is no excuse in the former case and not much in the latter, for if the dentist has not time to do all the work required in any given case, it is at least his duty to inform the patient that other work needs doing, so that she may avail herself of the services of another.

Is it not equally as wrong to use material which takes the shortest time to put in simply for the convenience of the operator and is it not adding to the remission when a temporary filling is used and the patient not apprised of the fact? If we are to have the confidence of our patients they must be treated justly and as intelligent beings. When plastics are used, such as the phosphates and gutta percha, the patient should know it and the reason why, so that they may give them attention when necessary. What can be said in excuse for the man who sends a patient away with several temporary fillings in the mouth without informing him how long such fillings usually last, why they are used, and when he should return to have them replaced with something more durable, or refilled with the same material as the case may demand.

And in regard to amalgam. I often wonder why so much of it is used, when it is not by any means the best material we have to work with. I think that most of us will acknowledge that far too much amalgam is used and by those, too, who might be expected to use better judgement. Among my patients I have come across many amalgam fillings in places where it ought not to be, it has looked both botchy and unsightly. In being asked why amalgam was used there I am often met with the answer that they did not know why it was used, they supposed the dentist was using what was best and no restriction was laid on the fees. Now there is evidently a wrong state of affairs existing in such cases, yet we must be ethical, the work has been done by a brother dentist, so we have to be very guarded in what is said.

I do not think amalgam ought to be used in cases where sufficient access can be had to a cavity and where the tooth is in a fit state to receive a gold filling, providing the patient can afford it. The cases where it is necessary to use amalgam in any of the anterior teeth, either superior or inferior, are seldom met with, and in nearly every case where it is used in those teeth it reflects but little credit on the operator and the profession at large. In nearly all cases I think the use of amalgam should be confined to the molars and then only when our patients can not afford the fee for gold or when the state of the tooth does not warrant its use.

It would seem that the reason so much amalgam is used is in many cases because of its ease of manipulation and the short time it takes to insert a filling, and also because there is more profit than in gold fillings. Many people are led to believe from the conversation of their dentists that it saves the teeth just as well as gold and that gold is only used because it looks better, and by people who want to pay a higher fee and have something different to that commonly used. Is not this all wrong? and should we not in conformity with the rapid strives our profession is making in both art and science, use that material which gives better artistic results and which calls for more art and skill in its use, even if the results were no better than are attained by the use of materials which do not call for so much skill in their manipulation, but when anything gives better results in the conservation of the teeth, there is all the more reason why it should be used in every possible case.

I said in the earlier part of my remarks that we ought to treat our patients in confidence. I believe in doing so. It is also our duty to give them all instruction in our power as well as work for them, and it is only by doing this that our profession will attain that prominence and success which all who love it are fighting for. It is more to people's advantage that they economise other ways than on the teeth, and by proper explanations and argument most of them see the force of such reasoning, but we can not expect to be so successful in this direction unless we combine aad work for the patient's best interest as well as our own. We ought to be consistent in all things and this applies to our charges for our work as well as the manner in which it is performed. I dare say you are all made to feel pretty tired by remarks our patients sometimes make, I know I am. Only a few weeks ago when I had completed some work for a patient and gave the fee charged, I was met with, "why do you charge so much as that, you only charged me so and so for the other amalgam filling you put in." I explained that the work I had formerly done was only a simple cavity and did not take half as long as that I had then done for her, and I had in justice to myself as well as to her, to charge in proportion. Her next remark was, "why, I never paid above a dollar for an amalgam filling and Dr. So and So will fill any old shell with amalgam for a dollar." Such cases are not rare and what a position it places one in, especially when the doctor referred to happens to be a member of our association and one who is looked upon as a professional brother in all respects. It is true that sometimes people talk in that way to get the advantage of you, but I am sorry to say that they often speak the truth.

Inconsistency among us only confounds our patients and works against our mutual success. My remarks about amalgam will apply in a sense to rubber plates and I am here led to ask the question, is there not too much rubber used as well as amalgam? Of course our patients pockets must be considered, but when a patient can afford the better material for the base of their plates let us by all means use it, for I think no one will deny that a gold plate properly constructed is far better than the best rubber work that can be done. Whether it is the coloring matter, the presence of mercury or the non-conductibility of rubber, that causes it to be so objectionable as a base in nearly all cases, and really injurious in some, has not yet been agreed upon, but all of us know, that in many cases rubber cannot be worn without a very flabby, inflamed condition of the mouth being the result, and such condition is always very much reduced and in most cases entirely so, when a metal base is substituted, which facts speak more than all the arguments in the world in favor of metals and porcelain as bases for artificial teeth.

It is no uncommon occurrence to come across cases in which the use of rubber has to be abandoned owing to the diseased condition of the mouth and a material more compatible with the health of the mouth substituted. The question comes, why is it that so much rubber is used? I think the reason is not far to find; it is cheap both to patient and dentist, it is so easy to manipulate, and I am inclined to believe that many well qualified men in other respects are sadly lacking in the ability necessary to successfully construct a metal plate. Rubber is very cheap, it is very easy to work and there is just as much or more profit in it as in the higher class work, and say some of our patients demand it, but is it used altogether because the people demand it? do they demand it? I do not think so. It is true that many people can not afford the higher class work, but the number of people who are wearing rubber work, who could well afford the best and would willingly pay for it were its numerous advantages and comforts pointed out to them, do not stand as creditable monuments to us as dentists, either as business men, or as men who are supposed and I hope are trying to give their patients the best possible advantages to be gained from our learning and knowledge.

I have found on conversing with patients that some think that gold is a thing of the past and that rubber is an improvement on it and is the best for plates, and that their dentists have in some cases told them so. It is not three months since I had a patient who had three rubber plates and could not wear them on account of their bulk. On asking him why he had not tried a metal plate, he said, he had not been advised to do so, in fact, one dentist told him he had better get rubber, as, if he could not wear it, he would not be out so much as if he got a gold plate. Certainly such talk and methods are not apt to be very successful in inspiring our patients with confidence. To be sure there are penurious individuals who always want the cheapest, but when such people have the advantages of the better material pointed out to them, they often leave themselves in our hands. It is, I feel sure, our duty to point out all advantages to our patients and endeavor to get them to have what is best for his or her particular case and not the cheapest.

Our practices should be governed by us and not by our patient. If we do not know what is best for them there is no use in them coming to us, and if we do not use our talents in our patient's best interest, we have no right to be practicing a profession on which so much that is of almost vital importance depends and which calls for not only the best skill at our command, but also for deep thinking and matured judgement. I believe our patients are very much what we make them, they are in our hands to mould and instruct properly and our patients have confidence in us only to the degree that our methods and acts inspire. A man can revel in a rubber plate and amalgam filling practice, in which only pockets on both sides are considered, or he can have a practice in which all his efforts are concentrated for the best service of his patients. Higher education is being talked of on all hands and we, as dentists, should not be behind in imparting the best and most liberal education possible to our patients and especially to the younger people and those who have charge of them. Anything that tends to elevate our profession and bring its best manipulative skill and judgement out in the interest of our patients should be resorted to especially when such methods are the best.

Such a mode of practice will educate our patients and in educating them and treating them honestly, we are most certainly paving the way to our own individual success and to our still higher attainment and elevation as progressive and scientific professional men.

A Remedy for Tender Feet.—A remedy for tender feet is cold water ; about two quarts ; two tablespoonfuls of ammonia ; one tablespoonful of bay rum. Sit with the feet immersed for ten minutes, gently throwing the water over the limbs upward to the knee. Then rub dry with a crash towel, and all the tired feeling is gone. This is good for a sponge bath also.—*The Journal of the American Medical Association.*

Alcohol and Tea.—Dr. Kraepolin, as a result of experiments upon the psychical effects of alcohol and tea, finds that the former substance in moderate doses hastens volitional acts, but does not hasten deliberation and association processes. The purely reasoning processes in fact are lessened by alcohol. Tea, on the other hand, does not hasten volitional acts, but does accelerate purely intellectual processes of association and reasoning.—*Medical Record.*

